# A single-nucleotide polymorphism C-724 /del in the proter region of the apolipoprotein M gene is associated with type 2 diabetes mellitus

**DOI:** 10.1186/s12944-016-0307-3

**Published:** 2016-08-30

**Authors:** Pu-Hong ZHANG, Jia-Lin GAO, Chun PU, Gang FENG, Li-Zhuo WANG, Li-Zhu HUANG, Yao ZHANG

**Affiliations:** 1 Department of Biochemistry and Molecular Biology, Wannan Medical College, 22 West Wenchang Road, Wuhu, 241002 People’s Republic of China; 2 Anhui Province Key Laboratory of Biological Macro-molecules Research, Wannan Medical College, Wuhu, People’s Republic of China; 3 Department of Clinical Laboratory, Yijishan Hospital of Wannan Medical College, Wuhu, People’s Republic of China; 4 Department of Endocrinology and Genetic Metabolism, Yijishan Hospital of Wannan Medical College, Wuhu, People’s Republic of China

**Keywords:** Apolipoproteins, Diabetes, Single-nucleotide polymorphism

## Abstract

**Background:**

Apolipoprotein M (apoM) was the carrier of the biologically active lipid mediator sphingosine-1-phospate in high density lipoprotein cholesterol (HDL-C) and played a critical role in formation and maturation of prebeta-HDL-C particles. The plasma apoM levels were decreased obviously in patients with type 2 diabetes mellitus (T2DM). A new single-nucleotide polymorphism (SNP) C-724del in apoM promoter was associated with a higher risk for coronary artery diseases (CAD) and myocardial infarction, could reduce promoter activities and apoM expression in vitro. The primary aim of the present case-controls study was to investigate the effect of apoM SNP C-724del on apoM expression in vivo and its association with T2DM susceptibility in an eastern Han Chinese cohort.

**Methods:**

Two hundred and fifty-nine T2DM patients and seventy-six healthy controls were included in this study. Amplifying DNA of apoM proximal promoter region including SNP C-724del by Real-Time Polymerase Chain Reaction (RT-PCR) and amplicons sequencing. The plasma apoM concentrations were assayed by enzyme linked immunosorbentassay (ELISA).

**Results:**

Four polymorphic sites, rs805297 (C-1065A), rs9404941 (T-855C), rs805296 (T-778C), C-724del were confirmed. rs805297 (C-1065A) and rs9404941 (T-855C) showed no statistical difference in allele frequencies and genotype distributions between T2DM patients and healthy controls just as previous studies. It’s worth noting that the difference of rs805296 (T-778C) between these two groups was not found in this study. In SNP C-724del, the frequency of del allele and mutant genotypes (del/del, C/del) were higher in T2DM patients compared with healthy controls (*p* = 0.035; *P* = 0.040, respectively), while the plasma apoM levels of C-724del mutant allele carriers compared with the wide-type homozygotes carriers were not statistically different in T2DM patients (18.20 ± 8.53 ng/uL vs 20.44 ± 10.21 ng/uL, *P* = 0.245).

**Conclusion:**

The polymorphism C-724del in the promoter region of the apoM gene could confer the risk of T2DM among eastern Han Chinese. Unfortunately, the lowing of plasma apoM levels of C-724del mutant allele carriers compared with the wide-type homozygotes carriers in T2DM patients was not statistically different in present study, so further researchs were needed by enlarging the sample.

## Background

ApoM gene coded for a 26 kDa protein and located on chromosome 6q21–q23 which was a high susceptibility region to T2DM in genome-wide linkage analyses [1–3]. It was mainly present in high density lipoprotein cholesterol (HDL-C) and lesser extent in low density lipoprotein cholesterol (LDL-C), very low density lipoprotein cholesterol (VLDL-C), and chylomicrons [1, 4]. ApoM was the carrier of the biologically active lipid mediator sphingosine-1-phospate in HDL-C, played a critical role in formation and maturation of prebeta-HDL-C particles which could promote intracellular cholesterol efflux [5–7]. Several studies have identified promoter variants of apoM were associated with CAD and T2DM. Xu et al. have reported that the allele C carries of apoM rs9404941 (T-855C) polymorphism have an increased risk for CAD in Chinese populations [8]. Wu et al. and Zhou et al. have found that apoM rs805296 (T-778C) polymorphism was significantly associated with type 1 diabetes mellitus (T1DM) and T2DM in northern chinese population [9, 10]. Studies on 17 SNPs of apoM showed that apoM SNPs rs805297 (C-1065A) was associated with T2DM duration and SNPs rs707922 (G-1837T) TT genotype significantly increased the total cholesterol (TC) and LDL levels in patients with T2DM [11]. However, the genetic marker was distinct in different area such as northern and southern of China [12, 13], so the relationship between apoM rs805296 (T-778C) and the susceptibility of T2DM was not found in the southern Chinese population [11]. A new SNP C-724del, in the proximal promoter of apoM, was significantly associated with a higher risk for CAD and myocardial infarction, obviously decreased promoter activities and apoM expression in vitro [14, 15]. The primary aim of the present case-controls study was to investigate the effect of SNP C-724del on apoM expressions and its association with T2DM susceptibility in an eastern Han Chinese cohort.

## Methods

### Subjects

Two hundred and fifty-nine patients (149 males and 110 females) who were diagnosed T2DM by the WHO criteria [fasting glucose ≥7.0 mmol/L (126 mg/dL) or 2-h glucose ≥11.1 mmol/L (200 mg/dL)] [16]. Exclusion criteria were hematologic neoplastic, hepatic, thyroid, cardiac, autoimmune diseases or non diabetic kidney disease. Seventy-six age- and gender-matched healthy people (40 males and 36 females) who were good general health, no significant past medical history, and documented normal fasting blood glucose and glucose intolerance were selected as the controls group in the present study. All T2DM patients and healthy controls subjects were from the First Affiliated Yijishan hospital with Wannan Medical College. This protocol was approved by the Medical Ethics Committee of the WanNan Medical College in China, and all participants were provided written informed consent. Blood pressure was measured on the right arm in a sitting position after 15 min rest with a standard mercury sphygmomanometer. All participants had an overnight fast and patients did not take their usual medication and insulin before blood sampling.

### Methods

Plasma creatinine (Cr), cystatin c (CYS-C), superoxide dismutase (SOD), glucose, lipid levels and concentrations of urinary albumin were determined with Hitachi 7600 biochemistry autoanalyzer (Hitachi, Tokyo, Japan). All above reagents were purchased from BeiJing LEADMAN BIOCHEMISTRY CO, LTD, China. HbA1c was measured by HPLC using a Bio-Rad Variant II analyzer (Bio-Rad Laboratories, Hercules, CA, USA). Plasma apoM concentrations were assayed using a commercial ELISA kit (Cloud-Clone Corp, Houston, TX, USA) according to the manufacturer’s instructions. The concentrations of apoM in the calibrator was determined using a standard of known apoM concentration. All samples were diluted 1:5000 and analyzed in duplicate. The range of the standard curve was 0.312 ~ 20 ng/mL.

### Amplifying DNA of apoM proximal promoter region including SNPs C724del by RT-PCR and amplicons sequencing

Detail sequence information of apoM and SNP ID number are publicly available (http://www.ncbi.nlm.nih.gov/SNP). According to the sequence, a pair of primers were designed by primer premier 5.0 software (Premier Company, Canada) as follows: forward primer: AGTCACTTGGTGCTATCC; reverse: primer: GTTGGTGTCAGGCAGAAT, and the total sequence was 580 bp. The primers were synthesized by Sangon Biotech Company (Shanghai, China). Operation of extracting DNA from peripheral blood sample of each subject should accord with the instructions from a commercial DNA extract Kit (TIANamp, Beijing, China). Taq DNA polymerase, dNTPs, Polymerase chain reaction (PCR) buffer, and MgCl_2_ were all included in TIANamp Taq PCR Mastermix Kit purchased from TIANamp (Beijing, China). PCR was performed as follows: less than 0.1 ug genomic DNA template, 12.5 ul 2 × Taq PCR Mastermix, 10 umol of each primer and add ddH_2_O to a final reaction volume of 25 μl. Thermal cycling was performed in Veriti® PCR Thermal cycler (Applied Biosystems, America). The cycling program consisted of 3 min of initial denaturation at 94 °C, followed by 30 cycles at 95 °C for 30 s (temperature transition rate 3.9 °C/s), 60 °C for 30 s (temperature transition rate 3.9 °C/s), and 72 °C for 30 s (temperature transition rate 3.9 °C/s). PCR products of DNA including SNPs rs805297 (C-1065A), rs9404941 (T-855C) and rs805296 (T-778C), C-724del were directly sequenced on an automatic sequencer from Applied Biosystems (Model 3730, BGI, Shanghai, China).

### Statistical analysis

Continuous variables were provided as mean ± SD and categorical variables were expressed as percentages. Comparisons of the general characteristics between different genotypes in two groups were statistically evaluated with SPSS statistical package version 16.0 (Chicago, IL, USA). Comparisons of the genotype distributions between the two groups were tested by the linear-by-linear association Chi-squared analyses. Allele frequencies, Hardy-Weinberg equilibrium of genotype distributions, odds ratios (ORs) and 95 % confidence intervals (CIs) were estimated by the Chi-squared analyses. Analyses of linkage disequilibrium (LD) between SNPs were determined by calculating pair-wise D’ and r2 statistics in unrelated individuals using the SHEsis software online (http://analysis.bio-x.cn/myAnalysis.php). Power calculations were performed by SAS software (power and sample size, version 3.1). Significance was established at a *P* value < 0.05.

## Results

### General characteristics of T2DM patients and healthy controls

Comparisons of the baseline characteristics and plasma parameters between T2DM patients (*n* = 259) and healthy controls (*n* = 76) are shown in Table 1. There were no statistical difference in age and sex between T2DM patients and healthy controls (*p* = 0.940 and *p* = 0.450, respectively). Lower HDL-C, apolipoproteinAI (apoA-I), apoM (20.17 ± 10.03 ng/uL vs 24.48 ± 11.45 ng/uL, *p* = 0.004) were seen in T2DM patients compared with those in healthy control.

**Table 1:** Clinical characteristics of healthy control and T2DM patients

	Healthy control *N* = 76	T2DM *N* = 259	*P*-value
N (male/femal)	40/36	149/110	0.450
AGE (yrs)	56.24 ± 8.59	56.33 ± 11.49	0.940
SBP (mmHg)	117.36 ± 12.79	137.71 ± 20.89	**0.000**
DBP (mmHg)	76.00 ± 7.79	83.44 ± 11.40	**0.000**
Cr (umol/L)	64.80 ± 14.61	75.03 ± 31.73	**0.000**
CYS-C (mg/L)	0.97 ± 0.21	1.17 ± 0.50	**0.000**
SOD (U/mL)	106.70 ± 10.69	82.01 ± 25.78	**0.000**
FPG (mmol/L)	5.36 ± 0.36	9.45 ± 3.33	**0.000**
TC (mmol/L)	4.26 ± 0.52	4.46 ± 1.22	**0.038**
TG (mmol/L)	1.18 ± 0.42	2.19 ± 1.89	**0.000**
HDL-C (mmol/L)	1.41 ± 0.20	1.21 ± 0.32	**0.000**
LDL-C (mmol/L)	2.31 ± 0.41	2.53 ± 0.80	**0.002**
apoA-I (g/L)	1.66 ± 0.26	1.29 ± 0.34	**0.000**
apoB (g/L)	0.75 ± 0.18	0.90 ± 0.33	**0.000**
LP (a) (mg/L)	131.51 ± 113.31	157.07 ± 191.36	0.269
apoM (ng/uL)	24.48 ± 11.45	20.17 ± 10.03	**0.004**

Data mean ± SD

*N* number, *M* male, *F* female, *SBP* systolic blood pressure, *DBP* diastolic blood pressure, *Cr* creatinine, *CYS-C* cystatin c, *SOD* superoxide dismutase, *FBG* fasting blood glucose, *TG* triglycerides, *TC* total cholesterol, *LDL-C* low-density lipoprotein cholesterol, *HDL-C* high-density lipoprotein cholesterol, *apo* apolipoprotein, *LP (a)* Lipoprotein (a)

### Allele frequencies and genotype distributions of apoM promoter in T2DM patients and healthy controls

**Fig. 1:**
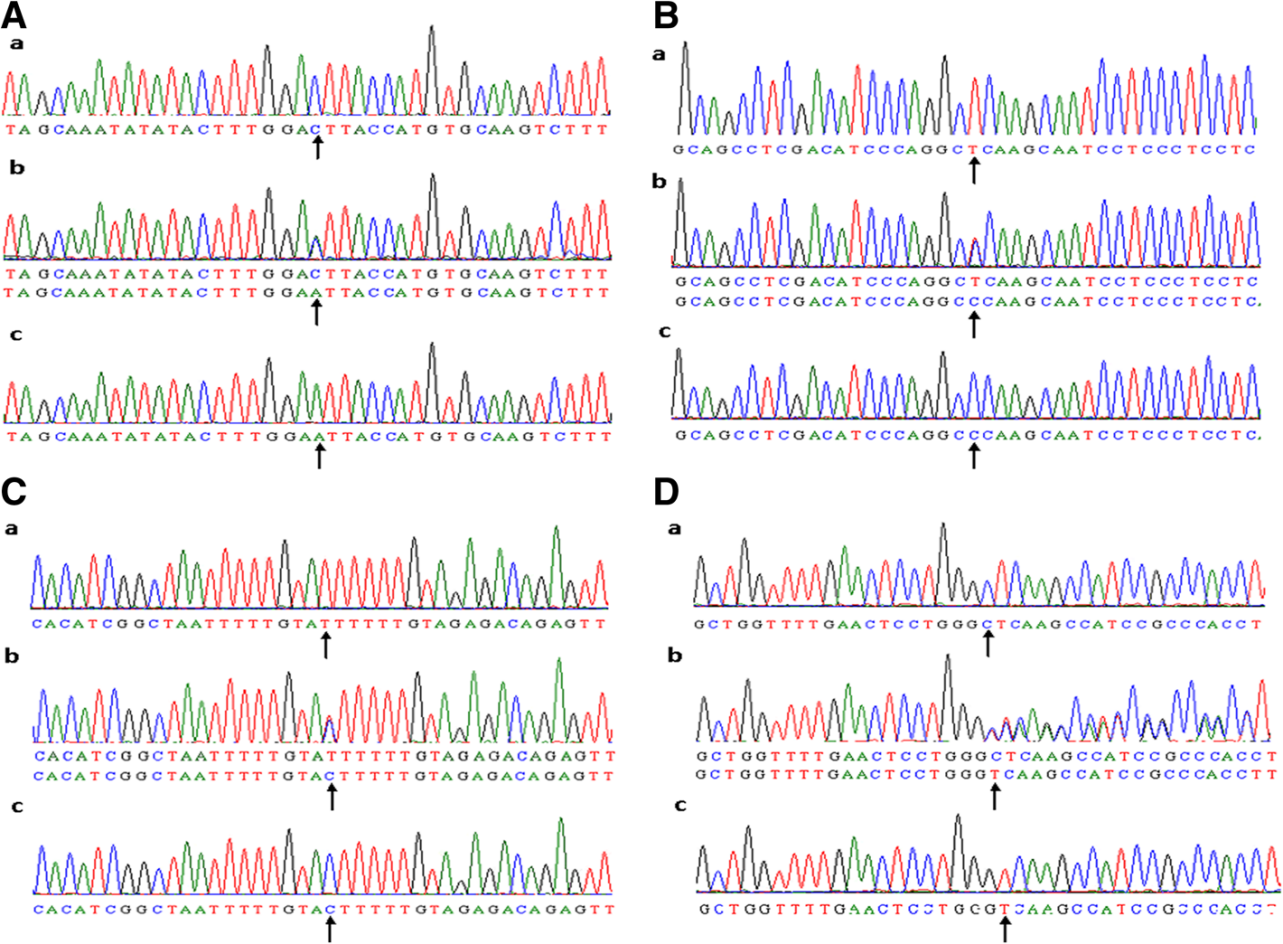
Four polymorphic sites, rs805297 (C-1065A), rs9404941 (T-855C), rs805296 (T-778C) and C-724del were confirmed in apoM promoter gene. **a**-*b* was a compound heterozygote for C-1065A. **a**-*a*, **a**-*c* were the sequence data of homozygotes for -1065C, -1065A, respectively. Similarly, **b**-*b*, **c**-*b*, **d**-*b* represent the compound heterozygote for T-855C, T-778C, C-724del, respectively. **b**-*a*, **c**-*a*, **d**-*a* were the sequence data of homozygotes for -855T, -778T, -724C, respectively. **b**-*c*, **c**-*c*, **d**-*c* were the sequence data of homozygotes for -855C, -778C, -724del, respectively. The *arrow* presented the mutation site

As showed in Table 2, C-1065A, T-855C, T-778C and C-724del were found in the present study (Fig. 1). Genotype distributions of these four SNPs in T2DM patients and healthy controls followed Hardy-Weinberg equilibrium (HWE, *P* > 0.05). C-1065A and T-855C showed no statistically significant difference in allele frequencies and genotype distributions between T2DM patients and healthy controls. Besides that, this study did not find the difference in allele frequencies and genotype distributions of SNP T-778C in these two groups. In T2DM patients, 212 patients (81.9 %) had the T/T genotype, 46 patients (17.8 %) had the T/C genotype and 1 patients (0.3 %) had the C/C genotype. While in healthy control, the rate were 73.7 %, 26.3 %, 0 %, respectively. The genotype distribution of SNP T-778C was not statistically different between T2DM patients and healthy controls (*P* = 0.146). The minor C allele frequency of SNP T-778C in the T2DM group was not significant different with healthy controls (9.3 % vs 13.2 %, *P* = 0.162). In SNP C-724del, the frequency of del allele was 6.4 % in T2DM patients and only 2.0 % in healthy controls (*P* = 0.035). In T2DM patients, 228 patients (88.0 %) had the C/C genotype, 29 patients (11.2 %) had the C /del genotype and 2 patients (0.8 %) had the del/del genotype. While in healthy controls, rates were 96.1 %, 3.9 %, 0 %, respectively. The genotype distribution was also significantly different between T2DM patients and healthy controls (*P* = 0.040).

**Table 2:** Allele frequencies and genotype distributions of apoM proximal promoter region in healthy control and T2DM patients

SNP ID	Allele	Allele frequency		Allele		Genotype								
		Control (%)	T2DM (%)	OR (95 % CI)	*P*-value	Healthy control (%frequencies)			T2DM (%frequencies)			*P*-value of HWE		*P*-value
						11	12	22	11	12	22	Control	T2DM	
C-1065A	C	67.1	67.8	1.030	0.879	36	30	10	115	121	23	0.356	0.265	0.878
rs805297	A	32.9	32.2	0.701 ~ 1.515		(47.4)	(39.5)	(13.1)	(44.4)	(46.7)	(8.9)			
T-855C	T	77.0	78.0	1.033	0.791	47	23	6	162	80	17	0.202	0.107	0.801
rs9404941	C	23.0	22.0	0.672 ~ 1.588		(61.8)	(30.3)	(7.9)	(62.5)	(30.9)	(6.6)			
T-778C	T	86.8	90.7	1.484	0.162	56	20	0	212	46	1	0.187	0.366	0.146
rs805296	C	13.2	9.3	0.851 ~ 2.587		(73.7)	(26.3)	(0)	(81.9)	(17.8)	(0.3)			
C-724del	C	98.0	93.6	3.379	**0.035**	73	3	0	228	29	2	0.861	0.323	**0.040**
	Del	2.0	6.4	1.022 ~ 11.176		(96.1)	(3.9)	(0.00)	(88.0)	(11.2)	(0.8)			

*P*-Value <0.05 was shown in bold

### Correlation between clinical characteristics and SNPs rs805297 (C-1065A), rs9404941 (T-855C), rs805296 (T-778C), C-724del in T2DM patients and healthy controls

Clinical characteristics and lipid levels of T2DM patients and healthy controls in relation to the genotypes of SNPs rs805297 (C-1065A), rs9404941 (T-855C), rs805296 (T-778C) and C-724del were showed in Tables 3, 4, 5, 6 respectively. In SNP C-724del, the mean TC level was lower in T2DM patients with mutant allele compared to the wide-type homozygotes carriers (*P* = 0.046).

**Table 3:** C-724del. Genotype and clinical characteristics of T2DM patients and healthy control

	Healthy control		T2DM		*P*-value	
	CC	C/- + −/−	CC	C/- + −/−	CC vs. (C/- + −/−) in healthy control	CC vs. (C/- + −/−) in T2DM patients
N (male/femal)	40/33	0/3	131/87	18/13	0.203	0.949
AGE (yrs)	52.67 ± 4.93	56.54 ± 7.68	56.26 ± 11.24	56.81 ± 13.14	0.392	0.830
SBP (mmHg)	110.00 ± 10.00	117.81 ± 13.15	137.93 ± 20.45	136.06 ± 24.20	0.314	0.641
DBP (mmHg)	74.00 ± 4.00	76.00 ± 8.07	83.64 ± 11.40	81.97 ± 11.44	0.672	0.444
DM duration (yrs)			6.55 ± 5.87	6.41 ± 5.61		0.895
HbA1c (%)			9.56 ± 2.50	9.58 ± 2.37		0.960
Cr (umol/L)	50.50 ± 9.03	65.01 ± 14.63	75.13 ± 32.65	74.30 ± 24.27	0.094	0.891
CYS-C (mg/L)	0.86 ± 0.10	0.98 ± 0.22	1.18 ± 0.51	1.15 ± .38	0.362	0.798
SOD (U/mL)	107.67 ± 15.50	106.87 ± 10.29	82.63 ± 25.96	77.42 ± 24.34	0.898	0.292
FPG (mmol/L)	5.19 ± 0.33	5.37 ± 0.37	9.43 ± 3.35	9.59 ± 3.22	0.409	0.807
TC (mmol/L)	4.20 ± 0.16	4.28 ± 0.52	4.52 ± 1.25	4.05 ± .93	0.779	**0.046**
TG (mmol/L)	0.64 ± 0.11	1.20 ± 0.41	2.22 ± 1.95	1.98 ± 1.35	**0.022**	0.503
HDL-C (mmol/L)	1.49 ± 0.11	1.42 ± 0.19	1.22 ± 0.31	1.19 ± 0.37	0.560	0.706
LDL-C (mmol/L)	2.42 ± 0.07	2.31 ± 0.41	2.55 ± 0.80	2.32 ± 0.74	0.125	0.119
ApoA-I (g/L)	1.63 ± 0.11	1.68 ± 0.26	1.30 ± 0.34	1.22 ± 0.32	0.748	0.210
ApoB (g/L)	0.71 ± 0.09	0.75 ± 0.18	0.91 ± 0.34	0.83 ± 0.29	0.706	0.228
LP (a) (mg/L)	210.37 ± 54.13	129.60 ± 115.45	157.20 ± 199.35	156.10 ± 119.13	0.234	0.976
apoM (ng/uL)	23.63 ± 8.80	24.93 ± 11.78	20.44 ± 10.21	18.20 ± 8.53	0.851	0.245

Data are means ± SD. *P*-Value <0.05 was shown in bold

**Table 4:** C1065A. Genotype and clinical characteristics of T2DM patients and healthy control

	Healthy control		T2DM		*P*-value	
	CC	CA + AA	CC	CA + AA	CC vs. (CA + AA) in healthy control	CC vs. (CA + AA) in T2DM patients
N (male/femal)	18/18	22/18	66/49	83/61	0.663	0.968
AGE (yrs)	55.00 ± 8.24	57.35 ± 8.84	57.50 ± 10.41	55.39 ± 12.23	0.236	0.141
SBP (mmHg)	118.31 ± 13.08	116.50 ± 12.62	139.84 ± 22.64	136.01 ± 19.29	0.542	0.142
DBP (mmHg)	76.39 ± 8.38	75.65 ± 7.32	85.13 ± 11.59	82.09 ± 11.10	0.683	**0.033**
DM duration (yrs)			6.32 ± 5.62	6.71 ± 6.00		0.600
HbA1c (%)			9.80 ± 2.51	9.37 ± 2.45		0.171
Cr (umol/L)	66.38 ± 16.05	63.39 ± 13.22	72.97 ± 30.90	76.68 ± 32.38	0.376	0.351
CYS-C (mg/L)	0.97 ± 0.20	0.97 ± 0.22	1.19 ± 0.53	1.16 ± 0.46	0.892	0.581
SOD (U/mL)	107.25 ± 10.84	106.20 ± 10.67	81.30 ± 19.61	82.58 ± 29.86	0.672	0.692
FPG (mmol/L)	5.28 ± 0.38	5.42 ± 0.33	9.92 ± 3.55	9.07 ± 3.10	0.087	**0.041**
TC (mmol/L)	4.18 ± 0.51	4.33 ± 0.53	4.49 ± 1.44	4.44 ± 1.02	0.206	0.767
TG (mmol/L)	1.16 ± 0.42	1.20 ± 0.42	2.19 ± 1.63	2.20 ± 2.08	0.647	0.957
HDL-C (mmol/L)	1.42 ± 0.20	1.41 ± 0.20	1.20 ± 0.31	1.22 ± 0.32	0.875	0.552
LDL-C (mmol/L)	2.24 ± 0.40	2.38 ± 0.41	2.53 ± 0.82	2.52 ± 0.78	0.142	0.936
ApoA-I (g/L)	1.64 ± 0.25	1.68 ± 0.28	1.27 ± 0.36	1.32 ± 0.31	0.500	0.240
ApoB (g/L)	0.72 ± 0.17	0.77 ± 0.19	0.89 ± 0.32	0.91 ± 0.35	0.232	0.597
LP (a) (mg/L)	117.72 ± 86.94	143.92 ± 132.57	156.66 ± 231.64	157.40 ± 152.60	0.317	0.975
apoM (ng/uL)	24.34 ± 11.02	24.62 ± 11.96	19.21 ± 8.46	20.93 ± 11.10	0.917	0.171

Data are means ± SD. *P*-Value <0.05 was shown in bold

**Table 5:** T855C. Genotype and clinical characteristics of T2DM patients and healthy control

	Healthy control		T2DM		*P*-value	
	TT	TC + CC	TT	TC + CC	TT vs. (TC + CC) in healthy control	TT vs. (TC + CC) in T2DM patients
N (male/femal)	21/26	19/10	90/72	59/38	0.077	0.406
AGE (yrs)	56.83 ± 9.37	55.28 ± 7.19	56.12 ± 12.14	56.68 ± 10.35	0.447	0.703
SBP (mmHg)	116.28 ± 13.45	119.10 ± 11.64	138.06 ± 20.66	137.13 ± 21.37	0.353	0.732
DBP (mmHg)	75.02 ± 7.37	77.59 ± 8.31	83.54 ± 11.54	83.27 ± 11.22	0.165	0.851
DM duration (yrs)			6.41 ± 5.84	6.74 ± 5.84		0.657
HbA1c (%)			9.61 ± 2.51	9.48 ± 2.44		0.663
Cr (umol/L)	62.20 ± 13.06	69.02 ± 16.17	74.91 ± 33.70	75.23 ± 28.29	**0.047**	0.938
CYS-C (mg/L)	0.95 ± 0.21	0.99 ± 0.21	1.18 ± 0.50	1.16 ± 0.48	0.418	0.789
SOD (U/mL)	106.64 ± 11.45	106.79 ± 9.53	83.19 ± 29.26	80.03 ± 18.55	0.952	0.341
FPG (mmol/L)	5.35 ± 0.36	5.36 ± 0.36	9.42 ± 3.29	9.50 ± 3.41	0.931	0.858
TC (mmol/L)	4.35 ± 0.48	4.12 ± 0.57	4.55 ± 1.36	4.32 ± .94	0.075	0.149
TG (mmol/L)	1.18 ± 0.39	1.18 ± 0.47	2.27 ± 2.03	2.07 ± 1.63	0.956	0.409
HDL-C (mmol/L)	1.42 ± 0.19	1.41 ± 0.22	1.21 ± 0.32	1.22 ± 0.31	0.940	0.810
LDL-C (mmol/L)	2.40 ± 0.37	2.18 ± 0.43	2.56 ± 0.83	2.47 ± 0.74	**0.023**	0.370
ApoA-I (g/L)	1.67 ± 0.26	1.64 ± 0.28	1.30 ± 0.34	1.28 ± 0.34	0.640	0.713
ApoB (g/L)	0.76 ± 0.18	0.72 ± 0.19	0.94 ± 0.37	0.85 ± 0.27	0.334	**0.034**
LP (a) (mg/L)	154.47 ± 128.70	94.30 ± 69.74	161.87 ± 219.55	149.06 ± 132.16	**0.023**	0.603
apoM (ng/uL)	26.63 ± 11.92	21.02 ± 9.87	20.55 ± 10.55	19.53 ± 9.13	**0.037**	0.427

Data are means ± SD. *P*-Value <0.05 was shown in bold

**Table 6:** T778C. Genotype and clinical characteristics of T2DM patients and healthy control

	Healthy control		T2DM		*P*-value	
	TT	TC + CC	TT	TC + CC	TT vs. (TC + CC) in healthy control	TT vs. (TC + CC) in T2DM patients
N (male/femal)	29/27	11/9	131/97	18/13	0.805	0.949
AGE (yrs)	55.21 ± 8.76	59.10 ± 7.57	56.12 ± 11.71	56.81 ± 13.34	0.082	0.764
SBP (mmHg)	118.73 ± 12.32	113.50 ± 13.58	136.83 ± 20.59	136.06 ± 24.20	0.117	0.851
DBP (mmHg)	76.57 ± 8.23	74.40 ± 6.34	83.11 ± 11.20	81.97 ± 11.44	0.288	0.598
DM duration (yrs)			6.49 ± 5.81	6.41 ± 5.61		0.943
HbA1c (%)			9.51 ± 2.48	9.58 ± 2.37		0.875
Cr (umol/L)	64.42 ± 13.46	65.88 ± 17.78	76.19 ± 33.37	74.30 ± 24.27	0.705	0.761
CYS-C (mg/L)	0.96 ± 0.19	0.98 ± 0.27	1.19 ± 0.53	1.15 ± .38	0.738	0.730
SOD (U/mL)	106.93 ± 10.18	106.05 ± 12.28	80.89 ± 20.97	77.42 ± 24.34	0.755	0.401
FPG (mmol/L)	5.38 ± 0.37	5.28 ± 0.32	9.43 ± 3.38	9.59 ± 3.22	0.249	0.813
TC (mmol/L)	4.24 ± 0.55	4.33 ± 0.45	4.36 ± 0.95	4.05 ± .93	0.525	0.097
TG (mmol/L)	1.16 ± 0.41	1.25 ± 0.45	2.14 ± 1.60	1.98 ± 1.35	0.401	0.596
HDL-C (mmol/L)	1.42 ± 0.20	1.40 ± 0.18	1.20 ± 0.31	1.19 ± 0.37	0.806	0.840
LDL-C (mmol/L)	2.30 ± 0.41	2.35 ± 0.41	2.49 ± 0.76	2.32 ± 0.74	0.596	0.224
ApoA-I (g/L)	1.65 ± 0.24	1.69 ± 0.34	1.28 ± 0.32	1.22 ± 0.32	0.625	0.350
ApoB (g/L)	0.75 ± 0.18	0.75 ± 0.18	0.89 ± 0.31	0.83 ± 0.29	0.950	0.318
LP (a) (mg/L)	121.14 ± 97.43	160.56 ± 148.32	160.62 ± 203.15	156.10 ± 119.13	0.183	0.904
apoM (ng/uL)	23.35 ± 10.91	27.68 ± 12.58	20.05 ± 10.21	18.20 ± 8.53	0.148	0.337

### Haplotype analyses

We estimated all possible haplotypes from the observed genotypes of rs805297 (C-1065A), rs9404941 (T-855C), rs805296 (T-778C) and C-724del by SHEsis software online (http://analysis.bio-x.cn/myAnalysis.php). The link-age disequilibrium analysis showed no obvious LD between any of two SNPs (Table 7). Haplotype (frequency > 3 %) derived from the four SNPs of apoM between T2DM and controlss was showed in Table 8. Among these four haplotypes, the haplotype T-T-del (-855-778-724), C-T-del (-1065-778-724) and C-T-del (-1065-855-724) were found to be associated with an increased risk of T2DM (OR = 3.268 95 % CI = 0.977 ~ 10.928; *P* = 0.040, OR = 3.375 95 % CI = 1.021 ~ 11.163; *P* = 0.035 and OR = 3.309 95 % CI = 0.998 ~ 10.978, *P* = 0.039, respectively). The power calculation showed that the study had 78.9 % power to detect differences of C-724del between T2DM and controls subjects at a significance level of 0.05.

**Table 7:** Linkage disequilibrium tests

Haplotype	rs9404941		rs805296		C-724del	
	D’	r^2^	D’	r^2^	D’	r^2^
rs805297	0.892	0.109	0.749	0.030	0.995	0.027
rs9404941	-	-	0.998	0.032	0.785	0.010
rs805296	-	-	-	-	0.940	0.006

**Table 8:** Association of apoM promoter haplotypes with T2DM

Haplotype		Haplotype frequency		OR (95 % CI)	*P*-value
		Cases	Controls		
H1	-855-778-724				
	T T del	0.061	0.019	3.268 (0.977 ~ 10.928)	**0.040**
H2	-1065-778-724				
	C T del	0.064	0.020	3.375 (1.021 ~ 11.163)	**0.035**
H3	-1065-855-724				
	C T del	0.062	0.020	3.309 (0.998 ~ 10.978)	**0.039**

Haplotype with frequency less than 3 % was pooled and not analyzed. *P*-Value <0.05 is shown in bold

## Discussion

In the present study, we performed genetic association analysis on apoM promoter SNPs rs805297 (C-1065A), rs9404941 (T-855C), rs805296 (T-778C) and C-724del in 259 T2DM patients and 76 healthy controls from an eastern Han Chinese population. It clearly demonstrated that C-724del polymorphism in the promoter region appeared to increase susceptibility to T2DM. However, the results of this study did not support an association between rs805296 (T-778C) polymorphism and T2DM susceptibility in eastern Han Chinese. Besides that, SNPs rs805297 (C-1065A) and rs9404941 (T-855C) had no significant difference in allele frequencies and genotype distributions between T2DM patients and healthy controls. Lower plasma apoM levels were also seen in T2DM patients than healthy controls in our study just as previous research [17]. However, the plasma apoM levels were not statistically different in T2DM patients with rs805297 (C-1065A), rs9404941 (T-855C), rs805296 (T-778C) and C-724del mutant allele compared to the wide-type homozygotes carriers respectively. Consistently, the haplotype analysis showed that the haplotype T-T-del (-855-778-724), C-T-del (-1065-778-724) and C-T-del (-1065-855-724) were associated with an increased risk for T2DM.

ApoM was a plasma apolipoprotein which was particularly present in HDL-C and to a lesser extent in TG-rich lipoproteins (TGRLP) and LDL-C [1]. It was required for prebeta-HDL-C formation and cholesterol efflux to HDL-C [7, 18, 19]. Besides that, apoM was associated with apoA-I, which was the main apolipoprotein in HDL and might be an independent predictor of apoA-I catabolism in overweight-obese, insulin resistant men [20, 21]. There were many evidences to suggest the involvement of apoM in the development of T2DM. Xu, et al. found that the apoM expression was reduced in alloxan-diabetic mice models [22], while Nojiri, et al. recently discovered the opposite result in streptozotocin-induced diabetic mice models [23]. Similarly, plasma apoM levels were lower in T2DM patients [17], our study also confirmed this result, so apoM might be a useful serum marker for the identification of T2DM, but the exact reasons were still unknown. ApoM was particularly present in HDL-C particles [4] and its expression was associated with liver X receptor (LXR) [24], fork head box A2 (Foxa2) [25, 26], liver receptor homolog-1 (LRH-1) [27] hepatic nuclear factor-1α (HNF-1α) [28], peroxisome proliferator-activated receptor beta/delta/gamma [29, 30], protein kinase C (PKC) [31], leptin and insulin [23, 32] et al., all of which were related to glucose metabolism. Animals and cell culture suggested that hyperglycemia could down-regulate apoM expression [33]. Moreover, apoM over expression might have a potential role in improving insulin resistance [34], further explain that modulating apoM expression might against insulin resistance in type 2 diabetes. Plasma levels of apoM were also decreased in maturity-onset diabetes of the young (MODY3) subjects, might attribute to the hepatocyte nuclear factor-1α (HNF-1α)-dependent impairment of apoM expression leaded by heterozygous HNF-1α mutations [35, 36].

This study found that the frequency of del allele in SNP C-724del were significantly increased in T2DM patients compared with healthy controls. In Zhou’ research, they reported that 290 individuals (88.0 %) had the C/C genotype, 18 individuals (5.8 %) had the C/del genotype, 1 individuals (0.3 %) had the del/del genotype and the frequency of del allele was 3.2 % in total 309 eastern Han Chinese controls [14]. The frequency of del allele were also significantly different between T2DM patients and above healthy controls (*P* = 0.013), both together, demonstrated that SNP C-724del was significantly associated with a higher risk for T2DM. In genetics, SNPs in the promoter region could affect the gene transcription and even protein expression. Zheng et al. found that constructs C-724del in apoM promoter region showed significant decreased promoter activity [14, 15]. That the expression of apoM was lower while the frequency of C-724del mutant allele in T2DM patients was higher compared with those healthy controls, suggested that this polymorphisms, in promoter region, might contribute to the down-regulation of apoM expression. Unfortunately, the lowing of plasma apoM levels of -C724del mutant allele carriers compared to the wide-type homozygotes carriers in T2DM patients was not statistically different (18.20 ± 8.53 ng/uL vs 20.44 ± 10.21 ng/uL, *P* = 0.245) in present study, so further researchs were needed by enlarging the sample.

ApoM SNP rs805396 (T-778C) was strongly associated with T1DM in both Han and Swedish populations, demonstrated that allele C of apoM SNP rs805396 (T-778C) may increase promoter activity and confer the risk susceptibility to the development of T1DM [9]. Moreover, apoM SNP rs805396 (T-778C) was also strongly associated with T2DM in Han [10], showed that apoM SNP rs805396 (T-778C) might be involved in the common pathogenesis of both T1DM and T2DM. It would have been ideal if the previous found that the SNP rs805296 (T-778C) were associated with T2DM in northern China were reproduced in this eastern Han Chinese. Unfortunately, the allele frequencies and genotype distributions between T2DM patients and healthy controls were not statistically different in this study just as previous research in Hong Kong Chinese population [11], the possible reason might be the difference in genotype of SNPs in different area of china [12, 13]. In addition, SNPs rs805297 (C-1065A) and rs9404941 (T-855C) had no significant difference in allele frequencies and genotype distributions between T2DM patients and healthy controls just as previous report [10]. It should be mentioned that the result of the present study needs further replications in other areas and races to avoid spurious associations which are common in genetic association researchs.

## Conclusions

In conclusion, the polymorphism C-724del in the promoter region of the apoM gene could confer the risk of T2DM among eastern Han Chinese. Unfortunately, the lowing of plasma apoM levels of C-724del mutant allele carriers compared to the wide-type homozygotes carriers in T2DM patients was not statistically different in present study, so further researchs were needed by enlarging the sample.

## Abbreviations

apo: Apolipoprotein
CAD: Coronary artery diseases
Cr: Creatinine
CYS-C: Cystatin C
DBP: Diastolic blood pressure
ELISA: Enzyme linked immunosorbent assay
FPG: Fasting blood-glucose
HDL-C: High-density lipoprotein cholesterol
LDL-C: Low-density lipoprotein cholesterol
Lp (a): Lipoprotein (a)
RT-PCR: Real-Time Polymerase Chain Reaction
SBP: Systolic blood pressure
SNP: Single-nucleotide polymorphism
SOD: Superoxide dismutase
T2DM: Type 2 diabetes mellitus
TC: Total cholesterol
TG: Triglyceride
VLDL: Very low-density lipoprotein cholesterol

## References

[1] Xu N, Dahlback B (1999). A novel human apolipoprotein (apoM). J Biol Chem.

[2] Xie T, Rowen L, Aguado B, Ahearn ME, Madan A, Qin S, Campbell RD, Hood L (2003). Analysis of the gene-dense major histocompatibility complex class III region and its comparison to mouse. Genome Res.

[3] Xiang K, Wang Y, Zheng T, Jia W, Li J, Chen L, Shen K, Wu S, Lin X, Zhang G (2004). Genome-wide search for type 2 diabetes/impaired glucose homeostasis susceptibility genes in the Chinese: significant linkage to chromosome 6q21-q23 and chromosome 1q21–q24. Diabetes.

[4] Dahlback B, Nielsen LB (2006). Apolipoprotein M--a novel player in high-density lipoprotein metabolism and atherosclerosis. Curr Opin Lipidol.

[5] Christoffersen C, Obinata H, Kumaraswamy SB, Galvani S, Ahnstrom J, Sevvana M, Egerer-Sieber C, Muller YA, Hla T, Nielsen LB, Dahlback B (2011). Endothelium-protective sphingosine-1-phosphate provided by HDL-associated apolipoprotein M. Proc Natl Acad Sci U S A.

[6] Christoffersen C, Nielsen LB, Axler O, Andersson A, Johnsen AH, Dahlback B (2006). Isolation and characterization of human apolipoprotein M-containing lipoproteins. J Lipid Res.

[7] Wolfrum C, Poy MN, Stoffel M (2005). Apolipoprotein M is required for prebeta-HDL formation and cholesterol efflux to HDL and protects against atherosclerosis. Nat Med.

[8] Xu WW, Zhang Y, Tang YB, Xu YL, Zhu HZ, Ferro A, Ji Y, Chen Q, Fan LM (2008). A genetic variant of apolipoprotein M increases susceptibility to coronary artery disease in a Chinese population. Clin Exp Pharmacol Physiol.

[9] Wu X, Niu N, Brismar K, Zhu X, Wang X, Efendic S, Du T, Liu Y, Gu HF, Liu Y (2009). Apolipoprotein M promoter polymorphisms alter promoter activity and confer the susceptibility to the development of type 1 diabetes. Clin Biochem.

[10] Niu N, Zhu X, Liu Y, Du T, Wang X, Chen D, Sun B, Gu HF, Liu Y (2007). Single nucleotide polymorphisms in the proximal promoter region of apolipoprotein M gene (apoM) confer the susceptibility to development of type 2 diabetes in Han Chinese. Diabetes Metab Res Rev.

[11] Zhou JW, Tsui SK, Ng MC, Geng H, Li SK, So WY, Ma RC, Wang Y, Tao Q, Chen ZY (2011). Apolipoprotein M gene (APOM) polymorphism modifies metabolic and disease traits in type 2 diabetes. PLoS One.

[12] Shi Y, Hu Z, Wu C, Dai J, Li H, Dong J, Wang M, Miao X, Zhou Y, Lu F (2011). A genome-wide association study identifies new susceptibility loci for non-cardia gastric cancer at 3q13.31 and 5p13.1. Nat Genet.

[13] Ren Q, Xiao J, Han X, Luo Y, Yang W, Ji L (2013). Rs290487 of TCF7L2 gene is not associated with type 2 diabetes in Chinese Han population: a case control study and meta-analysis. Exp Clin Endocrinol Diabetes.

[14] Guo H, Zhao XX, Zhang XJ, Chen W, Zhang J (2015). Functional study of -724I/D polymorphism in apolipoprotein M (apoM) gene promoter region and its association with myocardial infarction. Med Sci Monit.

[15] Zheng L, Luo G, Zhang J, Mu Q, Shi Y, Berggren-Soderlund M, Nilsson-Ehle P, Zhang X, Xu N (2014). Decreased activities of apolipoprotein m promoter are associated with the susceptibility to coronary artery diseases. Int J Med Sci.

[16] Alberti KG, Zimmet PZ (1998). Definition, diagnosis and classification of diabetes mellitus and its complications. Part 1: diagnosis and classification of diabetes mellitus provisional report of a WHO consultation. Diabet Med.

[17] Plomgaard P, Dullaart RP, de Vries R, Groen AK, Dahlback B, Nielsen LB (2009). Apolipoprotein M predicts pre-beta-HDL formation: studies in type 2 diabetic and nondiabetic subjects. J Intern Med.

[18] Wroblewska M (2011). The origin and metabolism of a nascent pre-beta high density lipoprotein involved in cellular cholesterol efflux. Acta Biochim Pol.

[19] Elsoe S, Christoffersen C, Luchoomun J, Turner S, Nielsen LB (2013). Apolipoprotein M promotes mobilization of cellular cholesterol in vivo. Biochim Biophys Acta.

[20] Faber K, Axler O, Dahlback B, Nielsen LB (2004). Characterization of apoM in normal and genetically modified mice. J Lipid Res.

[21] Ooi EM, Watts GF, Chan DC, Nielsen LB, Plomgaard P, Dahlback B, Barrett PH (2010). Association of apolipoprotein M with high-density lipoprotein kinetics in overweight-obese men. Atherosclerosis.

[22] Xu N, Nilsson-Ehle P, Ahren B (2006). Suppression of apolipoprotein M expression and secretion in alloxan-diabetic mouse: Partial reversal by insulin. Biochem Biophys Res Commun.

[23] Nojiri T, Kurano M, Tokuhara Y, Ohkubo S, Hara M, Ikeda H, Tsukamoto K, Yatomi Y (2014). Modulation of sphingosine-1-phosphate and apolipoprotein M levels in the plasma, liver and kidneys in streptozotocin-induced diabetic mice. J Diabetes Investig.

[24] Zhang X, Zhu Z, Luo G, Zheng L, Nilsson-Ehle P, Xu N (2008). Liver X receptor agonist downregulates hepatic apoM expression in vivo and in vitro. Biochem Biophys Res Commun.

[25] Hu YW, Zheng L, Wang Q, Zhong TY, Yu X, Bao J, Cao NN, Li B, Si-Tu B (2012). Vascular endothelial growth factor downregulates apolipoprotein M expression by inhibiting Foxa2 in a Nur77-dependent manner. Rejuvenation Res.

[26] Zhao JY, Hu YW, Li SF, Hu YR, Ma X, Wu SG, Wang YC, Gao JJ, Sha YH, Zheng L, Wang Q (2014). Dihydrocapsaicin down-regulates apoM expression through inhibiting Foxa2 expression and enhancing LXRalpha expression in HepG2 cells. Lipids Health Dis.

[27] Mosialou I, Zannis VI, Kardassis D (2010). Regulation of human apolipoprotein m gene expression by orphan and ligand-dependent nuclear receptors. J Biol Chem.

[28] Skupien J, Kepka G, Gorczynska-Kosiorz S, Gebska A, Klupa T, Wanic K, Nowak N, Borowiec M, Sieradzki J, Malecki MT (2007). Evaluation of Apolipoprotein M Serum Concentration as a Biomarker of HNF-1alpha MODY. Rev Diabet Stud.

[29] Luo G, Feng Y, Zhang J, Mu Q, Shi Y, Qin L, Zheng L, Berggren-Soderlund M, Nilsson-Ehle P, Zhang X, Xu N (2014). Rosiglitazone enhances apolipoprotein M (Apom) expression in rat’s liver. Int J Med Sci.

[30] Luo G, Shi Y, Zhang J, Mu Q, Qin L, Zheng L, Feng Y, Berggren-Soderlund M, Nilsson-Ehle P, Zhang X, Xu N (2014). Palmitic acid suppresses apolipoprotein M gene expression via the pathway of PPARbeta/delta in HepG2 cells. Biochem Biophys Res Commun.

[31] Yi-zhou Y, Bing C, Ming-qiu L, Wei W, Ru-xing W, Jun R, Liu-yan W, Zhao-hui J, Yong J, Guoqing J, Jian Z (2012). Dihydrotestosterone regulating apolipoprotein M expression mediates via protein kinase C in HepG2 cells. Lipids Health Dis.

[32] Luo G, Hurtig M, Zhang X, Nilsson-Ehle P, Xu N (2005). Leptin inhibits apolipoprotein M transcription and secretion in human hepatoma cell line, HepG2 cells. Biochim Biophys Acta.

[33] Zhang X, Jiang B, Luo G, Nilsson-Ehle P, Xu N (2007). Hyperglycemia down-regulates apolipoprotein M expression in vivo and in vitro. Biochim Biophys Acta.

[34] Zheng L, Feng Y, Shi Y, Zhang J, Mu Q, Qin L, Berggren-Soderlund M, Nilsson-Ehle P, Zhang X, Luo G, Xu N (2014). Intralipid decreases apolipoprotein M levels and insulin sensitivity in rats. PLoS One.

[35] Richter S, Shih DQ, Pearson ER, Wolfrum C, Fajans SS, Hattersley AT, Stoffel M (2003). Regulation of apolipoprotein M gene expression by MODY3 gene hepatocyte nuclear factor-1alpha: haploinsufficiency is associated with reduced serum apolipoprotein M levels. Diabetes.

[36] Mughal SA, Park R, Nowak N, Gloyn AL, Karpe F, Matile H, Malecki MT, McCarthy MI, Stoffel M, Owen KR (2013). Apolipoprotein M can discriminate HNF1A-MODY from Type 1 diabetes. Diabet Med.

